# Successful nedaplatin desensitization therapy in a patient with platinum-sensitive recurrent ovarian cancer: A case report and literature review

**DOI:** 10.1016/j.gore.2022.101065

**Published:** 2022-09-06

**Authors:** Tetsuya Kokabu, Kohei Aoyama, Yosuke Tarumi, Hisashi Kataoka, Kaori Yoriki, Taisuke Mori

**Affiliations:** Department of Obstetrics and Gynecology, Kyoto Prefectural University of Medicine, Graduate School of Medical Science, 465 Kajii-cho, Kawaramachi-Hirokoji, Kamigyo-ku, Kyoto 602-8566, Japan

**Keywords:** Desensitization, Hypersensitivity, Nedaplatin, Ovarian cancer, Platinum-sensitive

## Abstract

Desensitization protocols of platinum-based agents are recommended for patients with a history of hypersensitivity reaction (HSR). Herein, we report the first case of a successful desensitization therapy with nedaplatin after HSR to carboplatin and nedaplatin for platinum-sensitive recurrent ovarian cancer. A 53 year-old woman was diagnosed with stage IIIC serous carcinoma of the ovary and underwent primary debulking surgery followed by an adjuvant chemotherapy. The tumor relapsed 4 times in 10 years after the initial treatment, and platinum-based chemotherapy was performed on each occasion. HSR to carboplatin without and with desensitization protocol occurred during the 9th cycle of treatment and 2nd cycle of retreatment, respectively. Additionally, HSR to nedaplatin occurred during the 16th cycle of nedaplatin treatment. A four-step desensitization protocol with nedaplatin was conducted without occurrence of any severe adverse event. Nedaplatin desensitization regimen could be a new alternation for HSR to platinum-based agents.

## Highlights

• Platinum-based chemotherapy is the cornerstone of treatment for ovarian cancer.
• Hypersensitivity reaction (HSR) is a serious adverse reaction of anticancer drugs with anaphylactic features.
• HSR to platinum agents could lead to interruption of effective treatments for ovarian cancer, resulting in poor prognosis.
• We presented a case of successful nedaplatin desensitization therapy with a history of HSR to carboplatin and nedaplatin.
• The nedaplatin desensitization regimen could be an alternative for the patients with platinum sensitive ovarian cancer.

## 1 Introduction

Ovarian cancer has the highest mortality rate among gynecological malignancies (Onda et al., 2020). More than 50 % of patients with ovarian cancer are diagnosed at advanced stages. The best strategy for newly diagnosed epithelial ovarian cancer is complete debulking surgery, followed by platinum-based chemotherapy (Onda et al., 2020). However, unfortunately, about 70 % of ovarian cancers recur within 5 years despite multimodal treatments (Bartoletti et al., 2020).

Hypersensitivity reaction (HSR) is a serious adverse reaction of anticancer drugs with anaphylactic features (Çakmak et al., 2021). Although platinum-based agents play a crucial role in the treatment of ovarian cancer, the incidence of HSR increases following an increased frequency of administration of platinum-based agents (Bartoletti et al., 2020, Rose et al., 2003). The occurrence of HSR forces patients to discontinue their treatments in some cases. Desensitization is a well-known strategy against HSR with a high success rate (Cakmak et al., 2021, Castells et al., 2008, Hesterberg et al., 2009). However, patients who have had a history of HSR to one platinum agent often develop HSR to another (Michikami et al., 2013). Consequently, repeated HSR could lead to the failure of effective treatments for patients with platinum-sensitive recurrent (PSR) ovarian cancer. Nedaplatin, a platinum-based agents, is approved in several countries, and its efficacy and safety in patients with ovarian cancer has been reported (Michikami et al., 2013). Previous reports have demonstrated the efficacy of desensitization therapy with carboplatin, cisplatin, and oxaliplatin (Cakmak et al., 2021, Takase et al., 2015). However, to the best of our knowledge, nedaplatin desensitization therapy for patients with PSR ovarian cancer has never been reported. Herein, we report a successful desensitization therapy of nedaplatin in a PSR ovarian cancer patient with HSR to both carboplatin and nedaplatin, following maintenance therapy with olaparib.

## 2 Case report

A 53-year-old nulliparous woman with no medical history or known drug allergy presented to our hospital complaining of lower abdominal and inguinal pain in 2008. Transvaginal ultrasonography and pelvic magnetic resonance imaging (MRI) revealed bilateral polycystic tumors with solid components in the pelvic cavity. ^18^F-FDG positron emission tomography/computed tomography (PET/CT) showed a marked increase in FDG uptake in the solid cystic mass; however, there were no suspicious lymph node lesions, distant metastasis, or other primary lesions. No abnormal lesions were detected on upper gastrointestinal endoscopy or colonoscopy. A serum cancer antigen (CA)125 level of 315.5 U/mL was observed, but CA19-9 and carcinoembryonic antigen levels were not elevated. We diagnosed the patient with primary ovarian cancer based on the results of these examinations.

Primary debulking surgery, including abdominal total hysterectomy, bilateral salpingo-oophorectomy, pelvic and *para*-aortic lymph node dissection, omentectomy, and pelvic peritoneal biopsy, was performed. Intraoperative findings showed irregular masses in the Douglas’ pouch; there was no residual tumor after surgery. Histopathological evaluation revealed serous carcinoma of the ovary and metastases to the *para*-aortic lymph nodes that were not identified during pre-operative imaging examinations. Therefore, the patient was diagnosed with primary ovarian serous carcinoma, pT2cN1M0, stage IIIC (the International Federation of Gynecology and Obstetrics, 1988). There was no evidence of disease after 6 cycles of paclitaxel/carboplatin (TC) (paclitaxel 180 mg/m^2^ and carboplatin area under the curve [AUC] 6) as adjuvant chemotherapy.

**Fig. 1:**
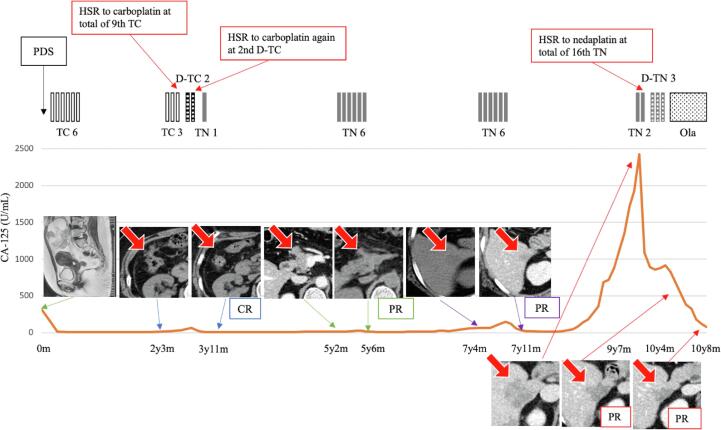
Clinical course and treatments The number of cycles is expressed in figures following TC or TN. Red arrows on CT images show dissemination, metastatic, and recurrent sites. Abbreviations: PDS, primary debulking surgery; TC, paclitaxel/carboplatin; D-TC, desensitization TC; TN, paclitaxel/nedaplatin; D-TN, desensitization TN; Ola, olaparib; HSR, hypersensitivity reaction; CR, complete response; PR, partial response.

However, twenty-seven months after the initial treatment, elevated serum CA-125 levels and peritoneal metastases were detected on CT. Although the patient received TC again, HSR to carboplatin occurred during the 9th treatment cycle. Therefore, carboplatin desensitization therapy was administered for subsequent chemotherapy. Unfortunately, HSR to carboplatin occurred again during the 2nd cycle of the desensitization treatment; therefore, paclitaxel/nedaplatin (TN) therapy (paclitaxel 175 mg/m^2^ and nedaplatin 80 mg/m^2^) was administered as a substituted for TC. The patient had a complete response after two cycles of TN therapy. TN therapy was repeatedly administered during each occasion of recurrence since then. Subsequently, the patient had a 4th relapse of ovarian cancer 9 years and 7 months after the initial treatment. The patient received TN therapy again, since 20 months has passed from the previous chemotherapy. Although a total of 15 cycles of TN therapy were completed without severe adverse events, the blood pressure and peripheral oxygen saturation suddenly dropped from 111/67 mmHg and 98 % to 98/56 mmHg and 94 %, respectively, with flushing, pharyngolaryngeal dysesthesia, dyspnea, and appearance of a rash after 30 min of initiation of intravenous drip infusion with nedaplatin in the 16th TN cycle; thus, the patient was diagnosed with HSR to nedaplatin. In response to it, a nedaplatin desensitization protocol was performed using a 4-step planned chemotherapy (Table 1). Consequently, the patient successfully underwent nedaplatin desensitization therapy combined with paclitaxel, without any severe adverse events, followed by olaparib maintenance therapy. The patient had a good clinical course without disease progression (Fig. 1).

**Table 1:** The protocol of desensitization therapy with nedaplatin.

				Volume(mL)	Solvent	Infusion time(min)
**Premedication before the infusion of nedaplatin**						
	Dexamethasone	9.9	mg	50	Normal saline	30
	Famotidine	20	mg			
	Granisetron hydrochloride	3	mg			
**Nedaplatin**						
1	1/1000 of the total dose			200	Normal saline	60
2	1/100 of the total dose			200	Normal saline	60
3	1/10 of the total dose			200	Normal saline	60
4	remainder of the dose			500	Normal saline	60

## 3 Discussion

Previous clinical trials have shown that platinum-containing chemotherapy for patients with PSR ovarian cancer is of significant benefit in progression free survival (PFS) (Bartoletti et al., 2020). Additionally, PFS was significantly improved by a maintenance therapy with poly ADP-ribose polymerase (PARP) inhibitors (Bartoletti et al., 2020). However, these trials also demonstrated that the administration of PARP inhibitors was recommended for patients with a partial or complete response to prior platinum-based chemotherapy (Bartoletti et al., 2020). Therefore, the importance of continuing platinum-based agents was reinforced in the treatment of patients with PSR ovarian cancer.

Clinicians often encounter HSR, which causes life-threatening conditions, to platinum-based agents in patients with ovarian cancer (Çakmak et al., 2021). The incidence of HSRs increases following an increase in the frequency of administration of platinum-based agents (Rose et al., 2003). For instance, the incidence of HSR to carboplatin and cisplatin is reported to have reached 44 % and 20 %, respectively (Ge et al., 2018, Li et al., 2014). Additionally, HSR to an alternate platinum-based agent tends to occur more frequently in patients with a history of HSR to a platinum agent (Michikami et al., 2013). The intradermal skin test could be a predictive marker of HSR to carboplatin (Confino-Cohen et al., 2005). However, more than 85 % of patients with positive skin test could receive desensitization without severe HSRs (Confino-Cohen et al., 2005). Moreover, the initially negative skin test might change to positive within 6 months after carboplatin exposure (Patil et al., 2012). Skin test may be unacceptable in clinical practice settings where carboplatin will subsequently be used within a narrow time interval. Thus, a skin test was not performed in this patient.

To date, several protocols for desensitization with platinum-based agents have achieved high success rates (Cakmak et al., 2021, Castells et al., 2008, Hesterberg et al., 2009). The survival outcome did not depend on the standard or desensitization administration in ovarian cancer (Park et al., 2020), indicating that desensitization therapy was acceptable for a clinical application in oncology fields. Table 2 shows previous reports on desensitization therapy with platinum agents for various cancers (Cakmak et al., 2021, Castells et al., 2008, Confino-Cohen et al., 2005, Hesterberg et al., 2009, Li et al., 2014, Rose et al., 2003, Takase et al., 2015, Vetter et al., 2019). The success rate of desensitization in at least one cycle was 87.5–100 %. Almost all desensitization regimens included H1/H2 blockers and/or steroids, such as dexamethasone, hydrocortisone, at various doses for the prevention of HSR. Regarding the dose of dexamethasone, 0–40 mg of dexamethasone was administered intravenously or orally prior to desensitization. However, there was no significant relationship between the completion rate and the premedication dose of dexamethasone. Thus, in the present study, premedication including dexamethasone (9.9 mg), famotidine (20 mg), and granisetron hydrochloride (3 mg), was administered before nedaplatin infusion. Considering patient safety in chemotherapy, it is known that there is a direct correlation between chemotherapy orders and errors related to chemotherapy. Errors were more likely to occur in patients who had received more than three injected chemotherapy drugs and had at least one dose modification (Weingart et al., 2018). However, some protocols require laborious and meticulous management during infusion. As a specific example, these protocols comprised up to 20 steps and the infusion rate had to be changed every 15–20 min (Cakmak et al., 2021, Castells et al., 2008, Hesterberg et al., 2009, Vetter et al., 2019). However, the four-step desensitization protocol was the most simplified schedule in desensitization protocols; it had a 95–98 % successful completion rate, and was suspended in 0–5 % of patients due to recurrence of HSR (Confino-Cohen et al., 2005, Li et al., 2014, Takase et al., 2015). Therefore, it was considered applicable to the nedaplatin desensitization therapy.

**Table 2:** The desensitization protocols and success rates.

Drug	Author	Year	No. of patients	Steps	Minimum concentration	Premedication (corticosteroid)	Duration (hour)	Success rate
Carboplatin	Çakmak ME, *et al.* [3]	2021	9	12–20	1:10000	DEX 40 mg p.o./i.v.	N/A	100 %
	Rose PG, et al. [4]	2003	33	4	1:1000	DEX 40 mg p.o./i.v.	16.5	88 %
	Castells MC, *et al*. [5]	2008	60	12	1:100	None	5.85	100 %
						DEX 20 mg i.v. + PTX		
	Hesterberg PE, *et al*. [6]	2009	13	8	1:10 (skin-test negative)	DEX 10 mg p.o.	6.35	97 %
			25	10	1:100 (skin-test positive)		11.05	
	Takase N, *et al*. [8]	2015	20	4	1:1000	DEX 24 mg i.v.	4	95 %
	Li Q, *et al*. [9]	2014	13	4	1:1	Hydrocortisone 50–100 mg i.v.	1.5	92 %
	Confino-Cohen R, *et al*. [11]	2005	20	4	1:1000	DEX 8–12 mg i.v.	6	95 %
	Vetter MH, *et al*. [14]	2019	36	16	Short 1:1	DEX 20 mg i.v.	1.5	87.5 % (including patients with cisplatin protocol)
					Standard 1:1		4.5	
					Prolonged 1:100		9	
Cisplatin	Castells MC, *et al*. [5]	2008	3	12	1:100	none	5.85	100 %
						DEX 20 mg i.v. + PTX		
	Li Q, *et al*. [9]	2014	5	4	1:1	Hydrocortisone50-100 mg i.v.	2.25	100 %
	Vetter MH, et al. [14]	2019	12	16	Short 1:1	DEX 20 mg i.v.	2.25	87.5 % (including patients with carboplatin protocol)
					Standard 1:1		5.25	
					Prolonged 1:100		16	
Abbreviations: DEX, dexamethasone; PTX, paclitaxel; p.o., per os; i.v., intravenous; N/A, not available.								

In the present case, the HSR to carboplatin occurred during the 9th cycle of carboplatin-based chemotherapy and 2nd cycle of the desensitization therapy with carboplatin. In comparison with cisplatin, nedaplatin has similar anticancer potency with lesser occurrence of nephrotoxicity and gastrointestinal events (Ge et al., 2018). Additionally, only 3.2–7.9 % of patients who had prior HSR to carboplatin developed HSR to nedaplatin, whereas 15 % of the patients developed HSR to cisplatin (Michikami et al., 2013). It was previously reported that TN therapy provided similar PFS with a better tolerance than TC for patients with PSR disease (Ge et al., 2018). Therefore, TN therapy was selected in subsequent treatments. Although the patient had received TN therapy on each occasion of recurrence, HSR to nedaplatin was unfortunately observed during the 16th cycle of TN therapy. We had to decide whether to continue with the same agent or to suspend the treatment. As the patient desired to continue subsequent chemotherapy, informed consent was obtained before the administration of nedaplatin desensitization therapy. Consequently, olaparib maintenance therapy was administered following completion of nedaplatin desensitization therapy, as originally scheduled. Six months have passed since olaparib maintenance therapy started, without clinical suspicion of recurrence.

## 4 Conclusion

To the best of our knowledge, there are no publications on nedaplatin desensitization therapy. Although further clinical studies are needed to evaluate the efficacy and safety of nedaplatin desensitization therapy, our case suggests that the nedaplatin desensitization regimen could be an alternative option for the continuation of crucial treatment for patients with PSR ovarian cancer.

Author contributions

Tetusya Kokabu collected basic information from the patient and image data, and wrote the manuscript; Kohei Aoyama, Yosuke Tarumi, Hisashi Kataoka, Kaori Yoriki contributed to the discussion and review/editing of the manuscript; Taisuke Mori supervised and made critical comments on the manuscript. All authors approved the final version of the manuscript.

## Declaration of Competing Interest

The authors declare that they have no known competing financial interests or personal relationships that could have appeared to influence the work reported in this paper.
